# Food insecurity among students living with HIV: Strengthening safety nets at the Nelson Mandela Metropolitan University, South Africa

**DOI:** 10.1080/17290376.2016.1218791

**Published:** 2016-08-17

**Authors:** L. Steenkamp, A. Goosen, D. Venter, M. Beeforth

**Affiliations:** ^a^ PhD (Dietetics), is a Research Associate at the HIV&AIDS Research Unit, Nelson Mandela Metropolitan University, Port Elizabeth, South Africa; ^b^ MCur (Advanced Primary Health Care), is a Head of Department at the Campus Health Service, Nelson Mandela Metropolitan University, Port Elizabeth, South Africa; ^c^ PhD, is a Statistical Consultant at the Unit for Statistical Consultation at the Nelson Mandela Metropolitan University, Port Elizabeth, South Africa; ^d^ BSc Dietetics, is a Postgraduate Student at the Department of Dietetics, Nelson Mandela Metropolitan University, Port Elizabeth, South Africa

**Keywords:** food insecurity, nutrition, hunger, HIV, higher education institutions, safety nets, l’insécurité alimentaire, l’alimentation, la faim, VIH, institutions d’enseignement supérieur, mesures de protection

## Abstract

The HIV prevalence in South Africa among students at higher education institutions (HEIs) in 2008 was reported to be 3.4%, with the highest HIV prevalence found in the Eastern Cape Province. Students at these facilities are also increasingly affected by socio-economic constraints that may impact on food security. Little is known about the impact of food insecurity on HIV-infected students in HEIs in South Africa. The purpose of this paper is to describe food insecurity and the nutritional status among HIV-infected students on the Nelson Mandela Metropolitan University campuses in South Africa, as well as current initiatives to strengthen the safety nets for food-insecure students. This descriptive, cross-sectional survey was conducted among a convenience sample of known HIV-infected, registered students (*n *= 63), older than 18 years of age and managed as part of the Campus Health Service antiretroviral therapy (ART) programme. Ethical approval for the study was obtained from the Research Ethics Committee (NMMU) and participants were included in the sample after providing written, informed consent. Findings indicate that food insecurity was common with more than 60% of the sample reporting food insecurity at the household level during the previous month. Of the sample, 51% were classified as being either overweight or obese. Although food insecurity did not contribute to weight loss in our sample, food-insecure students were more likely to consume inadequate amounts of vitamins and minerals, especially antioxidants that are important in supporting the immune system. Food insecurity has been identified as affecting the majority of HIV-infected students in this study, especially regarding their difficulty in accessing nutritious foods. As overweight and obesity also seem to threaten the health and future well-being of the students, appropriate management of the overweight individuals and those with obesity should be instituted in order to prevent the development of chronic diseases of lifestyle, thus allowing for a healthier more productive life. Current intervention strategies to strengthen food security have made inroads to improve access to healthier food options.

## Résumé

En Afrique du Sud, la prévalence du VIH parmi les étudiants du supérieur en 2008 est de 3,4% soit le taux le plus élevé dans la région de l’Eastern Cape. Les étudiants, dans ces établissements, sont également de plus en plus affectés par des contraintes socio-économiques qui peuvent affecter leur sécurité alimentaire. L’impact de l’insécurité alimentaire sur les étudiants, dans l’enseignement supérieur en Afrique du Sud, infectés par le VIH, est méconnu.

L’objectif de ce document est de décrire l’insécurité alimentaire et la situation nutritionnelle des étudiants infectés par le VIH, sur le campus de la Nelson Mandela Metropolitan University en Afrique du Sud, ainsi que les prises actuelles d’initiative pour renforcer la sécurité en ce qui concerne les étudiants en insécurité alimentaire.

Cette enquête transversale a été conduite sur un échantillon d’étudiants connus pour être infectés par le VIH, étudiants enregistrés (n = 63), âgés de plus de 18 ans et gérés dans le cadre du programme du Campus du service de santé de la thérapie antivirale (ART). L’approbation éthique pour mener l’étude a été obtenue par le Comité de recherche éthique (NMMU). Les participants ont été inclus dans l’échantillon après avoir fourni un consentement écrit.

Les conclusions indiquent que plus de 60% des personnes faisant partie de l’échantillon étaient en situation d’insécurité alimentaire au cours du mois précédant l’étude. 51% de cet échantillon ont été classés en surpoids ou obèses. Bien que l’insécurité alimentaire n’ait pas contribué à la perte de poids chez les personnes échantillonnées, les étudiants en insécurité alimentaire avaient plutôt tendance à consommer un taux inadéquat de vitamines et de minéraux, notamment des antioxydants importants dans la régulation du système immunitaire. Dans cette étude, l’insécurité alimentaire a été identifiée comme affectant la majorité des étudiants infectés par le VIH, notamment en ce qui concerne l’accès à une nourriture nutritive.

Le surpoids et l'obésité fragilisent également la santé et le bien-être futur des étudiants, une gestion adaptée des individus en surpoids doit être instituée pour prévenir le développement des maladies chroniques et ainsi pour une vie meilleure et plus saine. Les stratégies d’intervention actuelles pour renforcer la sécurité alimentaire ont fait des avancées pour améliorer l’accès à une alimentation saine et équilibré.

## Introduction

Food security, as defined in 1996 by the World Health Organization (WHO), is ‘when all people at all times have access to sufficient, safe, nutritious food to maintain a healthy and active life’ (WHO 2014). South Africa is described as a food-secure country at the national level, being a net exporter of agricultural products, with no major food shortages resulting from drought or environmental shocks (Koch 2011). However, household food insecurity is increasing in this country with poverty and unemployment limiting individuals’ purchasing power (Labadarios, Davids, Mchiza & Weir-Smith 2009).

A national survey among adults in South Africa showed that 29% of respondents reported that they ‘sometimes’ do not have enough food to eat and 9% reported ‘often’ not having enough food to eat (Sorsdahl, Slopen, Siefert, Seedat, Stein & Williams 2011). In the recent South African National Health and Nutrition Examination Survey (SANHANES-1) less than half of the sample (46%) was food secure, 28% reported to be at risk of hunger and 26% experienced hunger. The Eastern Cape and Limpopo Provinces had the highest prevalence of food insecurity with more than 30% of participants in both provinces reporting hunger (Shisana, Labadarios, Rehle, Simbayi, Zuma, Dhansay, *et al.* 2013).

Students at higher education institutions (HEIs) are also increasingly affected by socio-economic constraints. In 2005, the Human Sciences Research Council (HSRC) revealed that insufficient funds or lack of financial support should be considered the main reason for students dropping out and not completing their studies (Letseka, Cosser, Breier & Visser 2010). Insufficient funds do not only impact on students’ ability to pay for their studies, but also affects among others, food security. Munro, Quayle, Simpson and Barnsley (2013) recently showed that, despite more than a third of a sample of students at the University of Kwazulu-Natal (UKZN) receiving financial aid, 16% in that sample also reported some level of food insecurity, and almost 5% showed serious levels of vulnerability to food insecurity.

Apart from hunger and food insecurity threatening the well-being of South Africans, the spread of the human immunodeficiency virus (HIV) and acquired immunodeficiency syndrome (AIDS) remains a major concern with the 2012 HIV prevalence in South Africa being estimated at 12.2% (Shisana, Rehle, Simbayi, Zuma, Jooste, Zungu, *et al.* 2014). According to this report, the HIV incidence in females between the ages of 15 and 24 years, was more than four times higher than the incidence found in males in this age group. Female students at HEIs, being part of this age group, are therefore also at a higher risk of HIV transmission.

The HIV prevalence among students at HEIs in South Africa in 2008 was reported to be 3.4% (HEAIDS 2010) with the highest HIV prevalence found in the Eastern Cape (6.8%). In this study the poorer socio-economic bracket was independently associated with being HIV infected and transactional sex (in exchange for material gain) seems to be commonplace, leading to an increased HIV transmission risk. As statistical findings regarding sexual risk behaviour among students support an increase in the HIV infection rate at South African HEIs (Van Staden & Badenhorst 2009), the Higher Education & Training HIV/AIDS programme facilitates the implementation of a number of HIV prevention strategies. This ongoing programme assists HEIs to strengthen their response to the HIV and AIDS epidemic. Yet, in a recent survey by HEAIDS (Mbelle, Setswe, Sifunda, Mabaso & Maduna 2014), negative attitudes towards condom use were common despite a high awareness about HIV infection and transmission. It was also shown in this report that 29% of students do not have sufficient money for basics, such as food and clothes, emphasizing the vulnerability of a large proportion of students across campuses.

Apart from food insecurity potentially influencing HIV risk behaviour, hunger can also negatively impact on the cognitive abilities of individuals (Munro *et al*. 2013). Furthermore, within the HIV-infected population, patients with a compromised nutritional status displayed reduced verbal learning and memory (Dolan, Montagno, Wilkie, Aliabadi, Sullivan, Zahka, *et al.* 2003). Apart from the nutritional status potentially affecting HIV-infected students’ learning outcomes, HIV may also compromise their morbidity and mortality (Liu, Spiegelman, Semu, Hawkins, Chalamilla, Aveika, *et al.* 2011). To optimize health, well-being, and learning outcomes in students living with HIV, prevention and/or management of food insecurity in this vulnerable group and protecting their nutritional status should be considered a priority. There is, however, limited information available regarding the prevalence of food insecurity and the nutritional status of HIV-infected students in South Africa. As these aspects may eventually have a profound impact on their quality of life (Palermo, Rawat, Weiser & Kadiyala 2013; Thapa, Amatya, Pahari, Bam & Newman 2015) and possibly also on their learning outcomes, it is necessary to describe the prevalence of food insecurity and the nutritional status in HIV-infected students on campuses.

The purpose of this paper is to describe food insecurity and nutritional status among a sample of HIV-infected students at the Nelson Mandela Metropolitan University (NMMU) as well as the resulting initiatives to strengthen safety nets to manage food insecurity among these students.

## Methodology

This descriptive, cross-sectional survey was conducted among a convenience sample of known HIV-infected, registered students, older than 18 years of age and managed as part of the Campus Health Service antiretroviral therapy (ART) programme. All eligible participants were invited to participate and prospective clients who provided informed written consent were admitted to the sample between September 2012 and December 2013. Convenience sampling was used since the researchers had no alternative available in order to comply with ethical considerations. Ethical approval was obtained from the Research Ethics Committee (Human), NMMU (H12-RTI-HIV-004).

Individual-level food insecurity was determined by asking the participant five questions based on the Household Hunger Scale (Ballard, Coates, Swindale & Deitchler 2011). This standardized tool was developed by the Food and Nutrition Technical Assistance Project (FANTA) to enable users to measure food insecurity in developing countries with cross-cultural accuracy (Deitchler, Ballard, Swindale & Coates 2010). Questions were asked according to the experiences of respondents in the previous month. For the purpose of this study, three to five ‘yes’ answers were interpreted as participants being regularly exposed to hunger and thus classified as being food insecure. Two or less ‘yes’ answers implied that the participant was mostly food secure.

A registered, trained, dietician experienced in interviewing techniques recorded a meal-based dietary history to assess usual individual food intake as recalled by the patient from the previous month. This was analysed using Foodfinder version 3. Results of all the micronutrients were compared to gender-specific Dietary Reference Intakes (DRIs) as developed and implemented by the Institute of Medicine and expressed as a percentage. For the purpose of this study a micronutrient intake of less than 70% of the DRI was classified as inadequate.

A trained dietician using standardized techniques measured the weight of participants to the nearest 0.1 kg using a Tanita BC 543 calibrated scale. Participants were measured in light clothing and without shoes. Height measurements were obtained to the nearest 0.1 cm and were measured with a stadiometer without shoes. All measurements were taken twice and the average value was used if the two differed. Two persons were involved in data capturing in order to identify errors and correct or remove conflicting data entries. The Body Mass Index (BMI), a simple index of weight-for-height that is commonly used to classify underweight, overweight and obesity in adults, was used in this study. It is defined as the weight in kilograms divided by the square of the height in metres (kg/m^2^). For the purpose of this study, a ‘normal’ BMI range was 18.5–24.99 kg/m^2^. A BMI of less than 18.5 kg/m^2^ was considered to be ‘underweight’, a BMI of 25.00–29.99 kg/m^2^ was considered ‘overweight’ and more than 30 kg/m^2^ as ‘obese’ (WHO 2011).

Statistical analysis was done with the Statistica software package (version 10). Frequencies and percentages were used to present categorical data and means and standard deviations for the numerical data. Subgroups were compared using the Pearson Chi-squared test to test for statistically significant differences. Comparison of means was done using the *T*-test to determine statistical significance (*p *< .05).

## Results

Participants (*n *= 63) had a mean age of 23.98 years (SD**= 5.38) with 79% being female (*n *= 50). Those participants with a known date of diagnosis (*n *= 59) reported being HIV-infected for a median of 7 months (IQR**= 1.97–19.75). Almost half of the sample (49%; *n *= 30) was managed on ART.

Food insecurity was measured with five questions. The Cronbach’s alpha coefficient of 0.83 indicated excellent internal consistency between these factors. Food insecurity was common within the sample with 65–71% reporting food insecurity at the household level during the previous month (Table 1). At the individual level, it was found that a notable percentage reported food insecurity in terms of both the quantity (49%, question 3; 40%, question 5) and the quality of their diet (67%, question 4), with both aspects influenced by financial constraints (see Table 1).

**Table 1.** Food insecurity based on selected questions of the household hunger scale (*n *= 63).

Question	*n*	%
**Household level**		
1. Household ran out of money to buy food	41	65
2. Limited number of foods available in the house	45	71
**Individual level**		
3. Student cuts or skip meals	31	49
4. Student ate less than what they think is healthy due to financial constraints	42	67
5. Student was hungry at least once in the previous month due to financial constraints	25	40

Despite the high prevalence of food insecurity in the sample, participants had a median BMI of 25 kg/m^2^ (IQR**= 22.4–31.9) with 21% (*n *= 13) of the sample being categorized as ‘overweight’ and a further 30% (*n *= 19) classified as ‘obese’. Only 3% (*n *= 2) had a BMI less than 18.5 kg/m^2^, classified as ‘underweight’. Those participants classified as ‘obese’ reported a mean energy intake of 9965 kJ (SD**= 2432) versus 8862 kJ (SD**= 3332) in the ‘normal weight’ category. No significant association (Chi^2^ **= 2.8; *p *= .246) could be demonstrated between BMI and food insecurity with 25% (*n *= 10) of the sample classified as ‘obese’, also reporting food insecurity at household and individual level in the previous month.

However, results reflecting the quality of the diet were less positive. Findings confirmed that, despite a median intake of more than 100% of the DRI for all vitamins and minerals except vitamin D (see Table 2), a number of respondents consumed less than 70% of the DRI. More than 20% of the sample consumed less than 70% of the DRIs for vitamin A, selenium, vitamin E, vitamin C, and vitamin D as well as calcium and folate (see Table 3). Participants who experienced more food insecurity, showed a significantly reduced mean intake of calcium, magnesium, phosphorus, and zinc as well as thiamin and riboflavin compared to the food-secure group (see Table 2). Although participants’ BMI could therefore not be associated with the presence of food insecurity, more food-insecure students in the sample had a compromised vitamin and mineral intake.

**Table 2.** Daily micronutrient intake and percentage of DRI (*n *= 63) – Median and Mean values by food-secure group.

	Median		Mean (SD) % of DRI		Inferential	
Micronutrient	Daily intake	% of DRI	Food-secure Students (*n *= 27)	Food-insecure Students (*n *= 36)	*t*-Test *p*-value	Cohen’s *d*
Phosphorus	1098	189	226 (67)	178 (50)	.002	0.84
Thiamin	1.13	125	149 (53)	116 (48)	.011	0.67
Zinc	9.10	115	141 (63)	110 (30)	.002	0.66
Calcium	594	74	90 (37)	67 (34)	.014	0.64
Riboflavin	1.66	184	257 (171)	168 (119)	.017	0.62
Magnesium	269	97	116 (39)	98 (35)	.045	0.52
Selenium	42.1	94	127 (61)	99 (58)	.068	n/a
Iron	11.2	155	184 (79)	146 (88)	.083	n/a
Vitamin B6	1.61	146	169 (82)	138 (63)	.089	n/a
Vitamin C	69.0	115	174 (145)	119 (124)	.110	n/a
Folate	237	74	99 (53)	79 (57)	.134	n/a
Niacin	20.4	179	202 (96)	177 (71)	.231	n/a
Vitamin B12	3.50	175	320 (294)	254 (260)	.351	n/a
Vitamin D	4.64	46	57 (44)	48 (39)	.415	n/a
Vitamin E	10.8	90	114 (60)	105 (68)	.610	n/a
Vitamin A	768	149	213 (184)	208 (181)	.924	n/a

**Table 3.** Incidence of inadequate micronutrient intake (less than 70% of DRI) by the food-secure group.

Micronutrient	All (*n *= 63)		Food secure (*n *= 27)		Food insecure (*n *= 36)		Difference
	*n*	%	*n*	% A	*n*	% B	% B − % A
Calcium	29	46.0	9	33.3	20	55.6	22.2
Folate	29	46.0	9	33.3	20	55.6	22.2
Selenium	15	23.8	4	14.8	11	30.6	15.7
Vitamin C	22	34.9	7	25.9	15	41.7	15.7
Riboflavin	10	15.9	2	7.4	8	22.2	14.8
Vitamin B6	7	11.1	1	3.7	6	16.7	13.0
Thiamin	6	9.5	1	3.7	5	13.9	10.2
Vitamin D	45	71.4	18	66.7	27	75.0	8.3
Vitamin B12	9	14.3	3	11.1	6	16.7	5.6
Iron	4	6.3	1	3.7	3	8.3	4.6
Zinc	6	9.5	2	7.4	4	11.1	3.7
Magnesium	8	12.7	3	11.1	5	13.9	2.8
Phosphorus	1	1.6	0	0.0	1	2.8	2.8
Vitamin E	19	30.2	8	29.6	11	30.6	0.9
Niacin	4	6.3	2	7.4	2	5.6	−1.9
Vitamin A	13	20.6	6	22.2	7	19.4	−2.8

## Discussion

Increased access by students from previously disadvantaged communities to South African universities resulted in some of the socio-economic disparities found in the broader South African population also being reflected in HEI (Odhav 2009). Food insecurity is a growing concern and major threat to students. Approximately half of the participants in studies from two other South African universities reported food insecurity or hunger (Kassier & Veldman 2013; Van den Berg & Raubenheimer 2015). Most South African universities are responding to this threat by implementing safety nets, that is, the ‘No Student Hungry’ campaign at the Free State University (Department of Higher Education and Training 2011). Eligible students may apply for this food bursary or, alternatively, have the opportunity to apply for the National Student Financial Aid Scheme (NSFAS). Despite these safety nets, food insecurity still occurs and research (Kassier & Veldman 2013) shows that levels of hunger and food insecurity fluctuate during the semester, even among those with bursaries. That may complicate eligibility for food bursaries since students may only experience transient hunger for a few days at a time. Although the HIV prevalence on South African campuses is lower than that of the general population, HIV risk behaviour including transactional sex and substance abuse all play a role in the increased risk of HIV transmission among youth (Cluver, Orkin, Boyes, Gardner & Meinck 2011; Mbelle *et al*. 2014). Little, however, was known about the impact of food insecurity and the nutritional status of students living with HIV.

Results from this study at the NMMU indicate that more than 60% of HIV-infected students in the sample experienced some level of food insecurity during the month prior to the interview. This is substantially more than the 36% of NMMU students (*n *= 619) that reported skipping meals due to lack of funding according to data in a 2014 online survey (Gresse, Steenkamp & Pietersen 2015). Being a HIV-infected student would thus seem to increase the risk of food insecurity, which may create a vicious cycle since food insecurity impacts on both health and well-being.

At the NMMU various informal strategies within the campus health service were implemented over the past decade to address food insecurity among all students, not only those with HIV infection. These strategies were put in place with the specific objective not to stigmatize the HIV-infected population on campus, since it has been emphasized that efforts to reduced stigma should be incorporated into all HIV and AIDS programmes at tertiary institutions (Mbatha 2013). All students should thus have access to these programmes. During the last few years between 900 and 2000 students annually have been assisted with some form of nutrition support at the NMMU. However, specific programmes available to the HIV-infected student population include the following: All students who have tested HIV positive are enrolled into the campus health clinic’s HIV wellness programme which includes monthly visits to the clinic to consult with a professional nurse. The relevant student will, during these visits, have a full physical assessment which includes, among others, weight monitoring with the emphasis on preventing weight loss, as it is normally one of the key indicators pertaining to well-being of the HIV patient (Gripspoon & Mulligan 2003).

Interestingly, in this study at the NMMU, more than half of the sample was overweight despite the majority reporting periods of food insecurity. Overweight and obese HIV-infected patients like those found at the NMMU may be at an increased risk to develop other chronic health problems and metabolic disturbances and should be monitored closely for early signs and management of lipodystrophy (Abrahams, Maartens & Levitt, 2015). Energy needs during HIV infection is also still poorly understood and although recommendations by the WHO (2003) suggest a 10% increase in energy requirements over accepted levels during the asymptomatic phase, other researchers suggested that HIV-infected patients with viral suppression may have similar energy needs than those of normal subjects (Kosmiski 2011). It is evident that more research is needed to understand the associations between energy intake and weight changes in HIV-infected patients, especially in the presence of lipodystrophy. The high prevalence of overweight and obesity may also be linked to inactivity, a sedentary lifestyle, and poor dietary quality. Some students in the sample reported spending money on *vetkoek*, cheap chips, and sweets, which although more affordable, contain a high percentage of fat and refined carbohydrates and thus may contribute to overweight in food-insecure students. They would thus mostly consume refined carbohydrates/starches and only add more nutritious foods during periods in the month when they have access to food parcels or fresh produce. These unhealthy lifestyle habits have serious implications for the development of lipodystrophy and metabolic disturbances, such as hypertension and diabetes, later in life (Abrahams *et al.* 2015). Weight gain and not only weight loss should be added to monitoring criteria, as overweight and obesity also have health implications for the development of metabolic syndrome in the HIV-infected population (Rossouw, Botes & Conradie 2013).

Micronutrient deficiencies in HIV-infected persons are common (Balfour, Spaans, Fergusson, Huff, Mills, la Porte, *et al.* 2014). At the NMMU all HIV-infected students not eligible to receive ART are given monthly vitamin and mineral supplements to support the immune system. Students are also provided with monthly meal parcels containing non-perishable products to ensure, as far as possible, a daily healthy diet. Should any of the students be considered to be underweight they are provided with additional enriched porridge to ensure that no further weight loss occurs. Given the high median vitamin and mineral intake in the sample in this study, most eligible students are consuming the enriched meals provided. However, the fact that more than 20% of the sample reported inadequate intake of especially the vitamins and minerals supporting the immune system indicates that the nutrition programme should be extended to include nutrition counselling, during which affordable sources of dairy, whole wheat products, meat, seeds and lentils should be emphasized.

It has been established that food programmes for HIV-infected patients while on treatment may result in improved adherence to ART, fewer side effects and improved physical strength (Byron, Gillespie & Nangami 2008). At the NMMU, to further strengthen the food security situation, an agreement between the campus health service and the academic agriculture department was recently established where the agriculture students oversee a vegetable garden and chicken pens. The latter provide fresh vegetables, high-quality protein including eggs and chickens (slaughtered, packed and frozen) to ensure that the current carbohydrates and other non-perishable food items are supplemented on a monthly basis. This project is still in the infancy stage and has the vision and potential to grow and become a very substantial area of support to the nutrition project. Further needs including nutrition counselling and skills training of students with regard to food preparation should be developed and will further strengthen current approaches. Male students should also be targeted for these interventions since it was highlighted in a recent study that male students may be more at risk of food insecurity (Van den Berg & Raubenheimer 2015) and thus may be less equipped than females to make informed decisions on food selection and preparation.

When the HIV-positive students at the NMMU become eligible to receive ART they are transferred from the Wellness programme to the ART programme but remain on the nutrition project as described above. All of the above areas of care will remain in place until each student graduates and is transferred out to another ART facility.

The study was limited by the relatively small sample size; however, approximately half of known HIV-infected patients did participate. This indicated that most HIV-infected students are treated off-campus and that stigma should still be considered a huge problem in HIV-infected individuals. Efforts to address this should be increased during the annual voluntary counselling and testing campaigns.

In conclusion, food insecurity seems to affect the majority of HIV-infected students at this university and to a greater extent than HIV-uninfected students. It, however, did not contribute to weight loss in this sample since even overweight and obese individuals experienced food insecurity. This is most likely due to a poor dietary quality. As overweight and obesity threaten the health and future well-being of these young adults, it is important to monitor weight changes of HIV-infected students on ART programmes and provide them with both knowledge and access to better quality affordable food options. Current intervention strategies to strengthen food security have made inroads to improving access to healthier food options, but further development of these strategies is necessary to ensure improvement of the current situation. HIV-infected students are discharged from the programme after graduation or when they dropout. It is important to equip them with the necessary information in order to prevent the development of chronic diseases of lifestyle and thus allow for a healthier more productive life.
